# Supplementary material for: The Effect of Different Types of Fertilizers on the Growth of Cassava and the Fungal Community in Rhizosphere Soil
Source: J Fungi (Basel). 2025 Mar 19;11(3):235. doi: 10.3390/jof11030235 (PMC11943314; doi:10.3390/jof11030235)
Supplement: Supplementary file 1 [file jof-11-00235-s001.zip › jof-3468223-supplementary.pdf]

Supplementary Materials

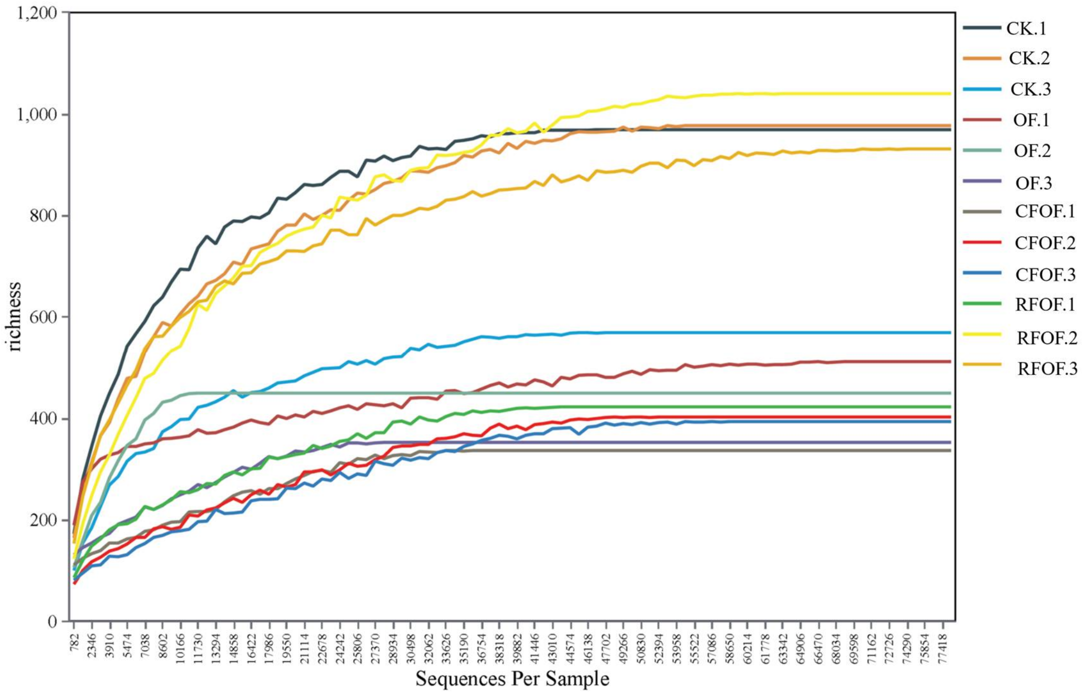

Figure S1. The rarefaction curves for fungal communities in different samples.

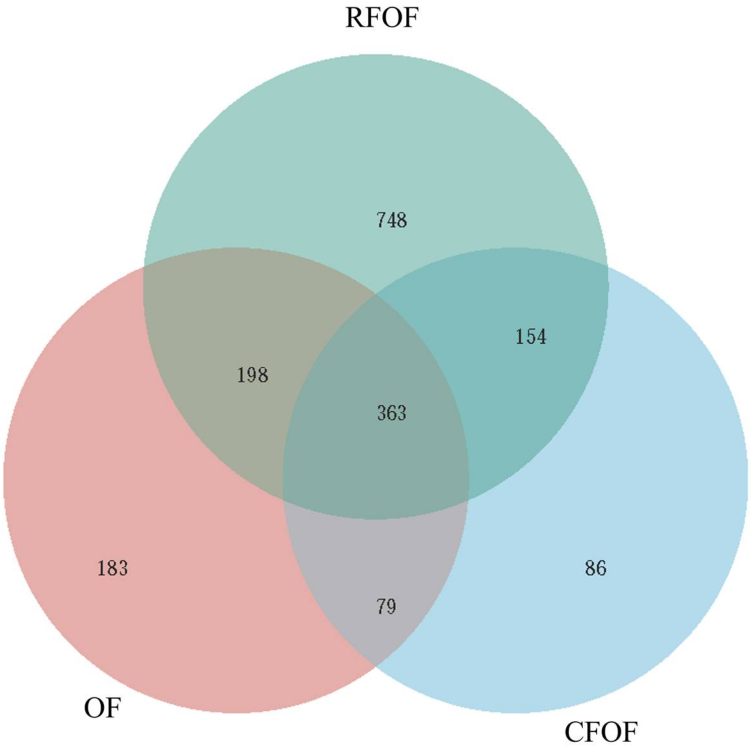

Figure S2. Venn diagram illustrates the shared and unique operational taxonomic units (OTUs) across different fertilization treatments.

Supplemental Table S1. The results of the high-throughput sequencing.

Sequencing	Sample ID	Raw reads	Clean reads	Clean tags	Q20 (%)	Q30 (%)	GC (%)
paired-end	CK.1	91189	91095	86032	99.4	97.3	48.0
paired-end	CK.2	86968	86877	82327	99.3	97.1	49.6
paired-end	CK.3	86263	86144	74803	99.2	97	44.5
paired-end	OF.1	92058	91965	85972	99.4	97.5	48.3
paired-end	OF.2	87421	87304	66610	99.6	97.9	53.5
paired-end	OF.3	84531	84410	77299	99.1	96.4	51.1
paired-end	CFOF.1	84129	84043	76504	99.3	97	51.8
paired-end	CFOF.2	89029	88905	80415	99.4	97.3	48.9
paired-end	CFOF.3	91549	91440	81547	99.4	97.4	49.2
paired-end	RFOF.1	89116	89016	80498	99.4	97.3	47.9
paired-end	RFOF.2	86499	86426	82456	99.4	97.5	53.6
paired-end	RFOF.3	91283	91198	84613	99.4	97.4	50.4
